# The Natural History of Pelvic Inflammations1Read by invitation before the Medical Society of the County of Erie, June 14, 1892.

**Published:** 1892-08

**Authors:** Joseph Price

**Affiliations:** Philadelphia; Fellow of the American Association of Obstetricians and Gynecologists; 500 North Twentieth Street


### THE NATURAL HISTORY OF PELVIC INFLAMMATIONS.[1]

By JOSEPH PRICE, M. D., Philadelphia.

Fellow of the American Association of Obstetricians and Gynecologists.

It is well to study the history of all affections, both from a clinical and a symptomatic standpoint, and afterward, when it is possible, reinforce and correct our knowledge by such pathological data as present themselves along the line of our investigation. Nowhere else has post-mortem examination served to clear the way to a correct clinical treatment more than in the disease under consideration.

1. Read by invitation before the Medical Society of the County of Erie, June 14, 1892.

The pathology of all the various forms of pelvic disease, though carefully inquired into and investigated several decades ago, until lately has rested upon a purely hypothetical basis. All the acute inflammatory lesions due to various causes and conditions, to which mention shall be devoted later, were loosely classed under a mythical pathology, based upon imagination rather than research, and designated cellulitis. Just what cellulitis is, and where we shall find it, is a perplexing question. A lately revised work on the diseases of women, which formerly anchored its faith and hope upon pelvic cellulitis, now confesses to have discovered that in many cases the so-called cellulitis had its origin in deep-seated peritoneal inflammation, and that many of the cases formerly designated as belonging to the cellulitic classification were, *undoubtedly, tubal* and *ovarian* in their *origin.* Now, the danger of "straddling" in this would-be differential diagnosis between what is cellulitis and what is not, lies in the fact that any purely symptomatic swelling of the pelvic cellular tissue is extremely rare, and the masses that are discovered, and termed infiltrated and congested connective tissue, are, in the vast majority of cases, not such tissue at all, but a conglomeration of structures, fused by inflammatory adhesions, whose destruction is not a simple cellular resolution, but has to do with many of the most important tissues of the body. What we want to remember from an anatomical standpoint is that which has been called periuterine deposits—phlegmons—cannot be located between the uterus and peritoneum, because of the absence here of sufficient cellular tissue to make it possible. As Bernutz says: "There is no cellular tissue lateral to the uterus, except just that which enters into the structure of the broad ligament." Now, as to broad ligament disease,—suppurative, I mean,—this is a question I believe I have a right to speak plainly upon. Within the last few years, or months, there has been a great deal of talk about broad ligament abscess, uncomplicated with tubal and ovarian disease. It is always a delicate matter to disagree and doubt absolute assertion, as I have myself tested, but I wish it clearly understood that my belief is one founded on my own and the broad experience of numbers of good men who work on my own lines, (or I, if you please, on theirs,) and do not take much for granted; so that when I say that in the experience of my own cases, and in those I have been either directly assisting or simply present, I have never seen an instance of simple, uncomplicated pelvic abscess, this statement must take value according to my experience. Now, when any observer with a limited experience beats the world in finding such cases, I must simply say to him, your observation has been incorrect, your investigation has not been thorough, your zeal is ahead of your knowledge. We all know how many anomalies are found the first day a student passes in the dissecting room, and this fever is abroad in surgery. Men who three years ago disputed the statement of then established operators, are now, in the language of our American or Kentucky game, "seeing them and going them one better" in all their work, and are out-heroding Herod in all that has been discovered or may be discoverable in the paths of gynecology and gynecological obstetrics. This is particularly true of the embryonic professors, whose feverish existence has not yet reached the normal temperature, but glows and frets and fears lest cooling shall not render their life problematical.

Now, in the most purulent inflammation discoverable in the broad ligament in tubal and ovarian disease, I beg you all to examine the parts removed, and see whether you will not discover that the ligament is itself intact. It is often folded on itself, glued together, and apparently fused with the abscess cavity, but careful dissection will free it, and we shall find that the ligament, except for inflammatory cloudiness due to adhesions, is in no way attacked by the pus deposit. And this is the invariable rule when removal is made *in toto.* This is why almost, without exception, all these pelvic tumors are removable. The broad ligament is the swing upon which is held the inflammatory neoplasm, and when intestinal and omental adhesions are loosed, the whole deposit may be lifted up and removed. Now, there is a reason why all this should be so, and what is it? Briefly, this: Almost without exception the *sources of inflammation come from the outside.* This is the key to the understanding of the whole matter. If the inflammation comes from without, its natural passage is through the uterus along the tubes, and, happily, here it is likely to stop, because the fimbriated peritoneal ending of the tube is prone to glue up, and so shut off the inflammatory matter from the general peritoneal cavity. But this is often accomplished only after the ovary is implicated, so that we find that ovarian disease is always secondary to tubal implication. Now, in ovarian abscess, the complications must necessarily be more extensive than in simple tubal disease, for obvious reasons. No matter what the primary cause of the inflammation may be, the lines of infection are most naturally those just referred to. If there is so deep and poisonous an infection as to implicate the uterine tissues subsequent to puerperal disease, and this goes on to penetrate the deep uterine structures, then, of course, we have no longer a trouble such as we have started out to describe, but a universal infecting focus, to-wit, the uterus itself. How rarely this must happen, even in the face of acute and virulent inflammations, is disclosed in the post-mortem room or on the operating table. Even in the retention of the placenta, which is by no means infrequent, and in the condition of subinvolution then present, this general septic condition, so much dwelt upon theoretically, is not often found. Just why this is true, I will not attempt to discuss here.

Now, as to the causation of pelvic inflammations in women. If these are understood and appreciated, there need be no serious difficulty in separating real from apparent disease. We are to remember, in the first place, that the history must put us on our guard as to the condition.

We are to enquire along the lines that are most likely to be followed by pelvic complications. Not only are we to seek natural but artificial causes. By artificial causes I mean those brought on not by art, but by clumsy skill, if I may be excused the paradox. *I am sure that many women are treated into pelvic disease.* In this connection we are to remember as practitioners and operators, that the simplest procedures apart from themselves may become serious under certain circumstances. The vaginal douche, for instance, whose immense value in pelvic congestions makes it an indispensable aid in their treatment, should never be used at the menstrual period, nor with too great force at any time. So used it may cause traumatic inflammation, and sow the seed for serious chronic ovarian and tubal disease. Other causes in the same line are the use of the sound, intrauterine applications, and the like. We are on the one hand advised to harbor and encourage such measures and instruments, but, if you please, as general practitioners, not as specialists; for with my own advice as a specialist, the safe path is to avoid inside tampering with peritoneal channels. Just how apparently simple interference with the interior of the uterus may give rise to such calamitous results as we sometimes see, is no more explicable, nor to be questioned, than the fact that the passage of a bougie into the male urethra occasionally kills, and without sepsis. No one with enough medical instruction to pass a first year's examination, will pass with impunity the sound into a bladder, neither ought every tyro in gynecology be made to think that the sound in gynecology is the key to diagnosis, nor the intrauterine application the safe path to success in the treatment of uterine disease. Along side of traumatic diseases of the operative sort must be placed those of disease and those of excessive venery. These, as causes, must enter into all our considerations, and be met with the necessary advice and treatment. In the history of the affections under consideration, the most teeming causes are gonorrhea, badly handled labors and abortions, and the accidents of menstruation. It may be well to consider this last class at once, for such cases are likely to be the ones to give us the most trouble on account of the age of the patient.

Young patients in gynecology are the bane of our work. Hence, we should have care to avoid anything operative, or in the way that gives the idea of unnecessary examination. Many early cases of pelvic inflammation in girls could be avoided, if their elders could be led to instruct them as to the necessary care to be taken at such times as they are menstruating, and the necessity for careful hygiene and the avoidance of the ruder forms of exercise, together with the fatigue of the ball room.

Badly handled labors and abortions must be reckoned as a fruitful cause of pelvic and inflammatory diseases of the ovaries and tubes. Hence, in making up our minds as to the presence or absence of disease previous to examination, we must inquire about all pregnancies, abortions and labors, and get exact histories of all these. In this connection—the management of labors—excessive douching at the puerperal period with excessively strong, irritating solutions, must give us reason to seek an irritating cause that may give us much trouble. We must inquire as to the use of instruments at labor, and to the after history, with reference to discharges. We must look after the retention of the placenta, and long continued bleeding, for all of these are competent to produce trouble along the channels already designated. The causal relation of gonorrhea in the production of the pelvic diseases of women, must always be considered in its true light. I find those who have reason apart from scientific data, most apt to question the casual relation of the two conditions. With me, from an enormous clinical experience and operative observation, it is an axiom, and needs little or no demonstration. The virus is so poisonous, and the channels are so patulous, that the wonder is that more women are not infected. The history is so often direct, following neither abortion nor puerperal complications, that it were almost a work of supererogation to insist upon it argumentatively. So much, then, for the causation.

The diagnosis, apart from the history, must rest upon digital examination. The presence of lateral masses in the pelvis, *our old cellulitic deposit*, must give us our point of inquiry. First, the uterus should be differentiated, after which, taking this as our objective point, all other relations are to be decided. The presence of a well-defined groove between the lateral masses and the uterus, points invariably to pelvic inflammatory deposit in the tubes and ovaries. The patient may suffer intensely on one side, and the disease be present on the other, hence, both sides should be examined, and the subjective sensations of the woman not be left to decide the point of the disease. Again, there may be exceedingly marked disease, and the ovarian and tubal complications small. Here it is well to remember that intestinal complications are often at the bottom of the size of the tumor, so that when adhesions are all freed, the mass that is to be removed is insignificant in its appearance, though examination made by floating the mass will disclose the multiplicity of the adhesions and their density. Another form of disease is the tubercular variety of ovarian inflammation. This is a most puzzling condition to deal with. We may examine and find what are apparently well-defined masses, but, on operating, discover nothing apparently justifying removal. Hence, in these cases, where it would seem that our diagnosis is at fault, as proven by operation, it is always advisable carefully to examine the appendages, to discover whether this condition does not obtain. In all the forms of inflammation under consideration, it is the tendency for the inflammation to be deposited in multiple locations. I mean that the abscess cavity is not apt to be single, but to consist of two or many pockets. This is due both to inflammatory strictures in the tube and to the different structures encountered in the inflammatory process. Hence, it is necessary to deal with all pelvic deposits as we know they must exist anatomically, taking into consideration their cause and the structures involved, and not treat them as hypothetical quantities under uncertain processes. If we consider the suppurating inflamed tube as an offending organ, quite as much so as the retained placenta, we cannot go astray in its treatment. It is just as logical to treat the remaining *débris* of labor as a benign and inoffensive residue as to look at this internal disease as harmless; indeed, it is much more rational, for the one has some chance of escape, the other none. In this superficial and necessarily hasty consideration of my subject, I have, no doubt, passed over some points of interest. Whatever such has been omitted, or left obscure, or appears to be incorrect, I trust, will receive open discussion, free and unrestrained, for the truth is not to be hid nor patched. The surgery of the abdomen must stand on its logic and its results. If these are bad, it must fail. If these are unsubstantial, it is worthless.

500 North Twentieth Street.

---

